# Sarcopenia and peripheral arterial disease: a systematic review

**DOI:** 10.1002/jcsm.12587

**Published:** 2020-07-10

**Authors:** Mégane Pizzimenti, Alain Meyer, Anne‐Laure Charles, Margherita Giannini, Nabil Chakfé, Anne Lejay, Bernard Geny

**Affiliations:** ^1^ FMTS, Department of Physiology, EA3072 Mitochondria, Oxidative Stress and Muscular Protection University of Strasbourg Strasbourg France; ^2^ Department of Physiology and Functional Explorations University Hospital of Strasbourg Strasbourg France; ^3^ Department of Vascular Surgery and Kidney Transplantation University Hospital of Strasbourg Strasbourg France

**Keywords:** Sarcopenia, Peripheral arterial disease, Pathological pathways, Oxidative stress, Inflammation, Mitochondrial function, Exercise training

## Abstract

**Background:**

Patients with lower extremity peripheral arterial disease (PAD) and sarcopenia are a population at risk requiring specific and targeted care. The aim of this review is to gather all relevant studies associating sarcopenia and PAD and to identify the underlying pathophysiological mechanisms as well as potential therapeutic strategies to improve skeletal muscle function.

**Methods:**

A systematic review was carried out following the recommendations of the Preferred Reporting Items for Systematic Reviews and Meta‐Analyses (PRISMA).

**Results:**

Data extraction allowed the evaluation of 140 publications; 87 met the inclusion criteria; of which 79 were included in the final review, reporting sufficient data for epidemiological and diagnostic criteria, mechanical analysis, and therapeutic approaches. Epidemiological analysis and diagnostic criteria were based on 18 studies following 2362 PAD patients [31.39% (SD 7.61) women], aged 72.42 (SD 2.84); sarcopenia was present in 34.63% (SD 12.86) of the patients. Mechanical and pathway analysis were based on five animal studies and 29 clinical reports, showing significantly altered muscle strength and function in 1352 PAD patients [26.49% (SD 17.32) women], aged 67.67 (SD 5.14) years; impaired muscle histology in 192 PAD patients (9.2% (SD 11.22) women), aged 64.3 (SD 0.99) years; +58.63% (SD 25.48) of oxidative stress in 69 PAD patients [16.96% (SD 8.10) women], aged 63.17 (SD 1.43) years; mitochondriopathy in 153 PAD patients [29.39% (SD 28.27) women], aged 63.50 (SD 1.83) years; +15.58% (SD 7.41) of inflammation in 900 PAD patients [40.77% (SD 3.71) women], aged 74.88 (SD 2.76) years; and altered signalling pathways in 51 PAD patients [34.45% (SD 32.23) women], aged 72.25 (SD 5.25) years. Therapeutic approaches analysis was based on seven animal studies and 21 clinical reports. In total, 884 patients followed an exercise therapy, and 18 received an angiogenesis treatment; 30.84% (SD 17.74) were women. Mean ages of patients studied were 66.85 (SD 3.96).

**Conclusions:**

Sarcopenia and lower extremity PAD have musculoskeletal consequences that directly impair patients' quality of life and prognosis. Although PAD is primarily a vascular disease, all etiological factors of sarcopenia identified so far are present in PAD. Indeed, both sarcopenia and PAD are accompanied by oxidative stress, skeletal muscle mitochondrial impairments, inflammation, inhibition of specific pathways regulating muscle synthesis or protection (i.e. IGF‐1, RISK, and SAFE), and activation of molecules associated with muscle degradation. To date, besides revascularization, the best therapeutic strategy includes exercise, but approaches targeting the underlying mechanisms still deserve further studies.

## Introduction

Cardiovascular diseases are a major cause of death around the globe, with a prevalence gradually increasing as life expectancy rises.^1^ Among them, lower extremity peripheral arterial disease (PAD) is defined by a reduction of or an obstruction to blood flow in the arteries, with symptoms ranging from intermittent claudication to critical limb ischemia (CLI), characterized by rest pain and/or ulcers.^2^, ^3^, ^4^

Peripheral arterial disease is commonly accompanied by musculoskeletal abnormalities including generalized loss of skeletal muscle mass, strength, and physical performance—also called sarcopenia.^5^, ^6^ Both PAD and sarcopenia can run in parallel, many patients with PAD being also diagnosed with a sarcopenic condition (and probably even more are undiagnosed). In addition to worsen the loss of muscle mass and function in a vicious circle, sarcopenia further aggravates patients' quality of life and prognosis.^7^ It is therefore essential to have a better understanding on how PAD and sarcopenia may impair skeletal muscle.

This systematic review resulted from the need to raise awareness on the importance of diagnosing sarcopenia in daily practice, and to better understand the pathological mechanisms underlying sarcopenia and PAD that might open the way towards new therapeutic advances. Therefore, the aim of this article is to gather all the relevant studies associating sarcopenia and PAD. To this end, the proposed systematic review will answer the following questions:

1. Epidemiological data: is sarcopenia a rare condition in patients with PAD?
2. Diagnostic criteria: how to diagnose sarcopenia and is sarcopenia a factor of poor prognosis for patients with PAD?
3. Mechanical analysis: how does sarcopenia affect skeletal muscle?
4. Therapeutic approaches: can we reverse the sarcopenic condition in patients with PAD?

## Methods

### Systematic review of the literature

A systematic review was performed following previously published Preferred Reporting Items for Systematic Reviews and Meta‐Analyses (PRISMA) guidelines.^8^

### Eligibility criteria

Throughout the process of literature selection, clear inclusion and exclusion criteria were followed. Studies included were full text English or French publications without any chronological limit. All primary research studies reporting a link between sarcopenia and PAD were included. Studies not eligible for inclusion were reviews, letters, editorials, comments, book chapters, and studies focusing on other diseases. The main outcomes of interest were the presence of epidemiological data, mechanical data, and therapeutic data focusing on skeletal muscle aberrations and PAD. Studies that listed patient characteristics, assessment method of sarcopenia, duration of follow‐up, and prognostic outcome were considered to have epidemiological significance. Studies that listed mechanistic pathways leading to muscular defects in PAD were considered to have mechanical significance. Studies that listed therapies targeting skeletal muscle for PAD patients were considered to have therapeutic significant.

### Information sources and search strategy

The PubMed database was analysed with a combined strategy using the subject heading terms (‘peripheral artery disease’ OR ‘peripheral arterial disease’ OR ‘critical limb ischemia’ OR ‘chronic limb‐threatening ischemia’) AND (‘sarcopenia’ OR ‘muscle atrophy’ OR ‘amyotrophy’ OR ‘muscle strength’ OR ‘muscle loss’ OR ‘muscle dysfunction’). All titles and abstracts collected from the search strategy were screened for relevance. When a relevant article was found, full text articles were retrieved. Studies that did not meet the inclusion criteria were excluded. The publications of the reference lists of included studies were searched and scanned for other potentially relevant studies. The full text of all potentially relevant articles was obtained and reviewed for eligibility.

### Study records and data items

Data were extracted using a standardized form. This was done in duplicate to increase accuracy and to reduce measurement bias. Data extracted included study characteristics (year of publication, study design, population, and parameters determined) and particularly skeletal muscle characteristics (type of muscle, measurement method of muscle mass and strength) and main results.

## Results

**FIGURE 1:**
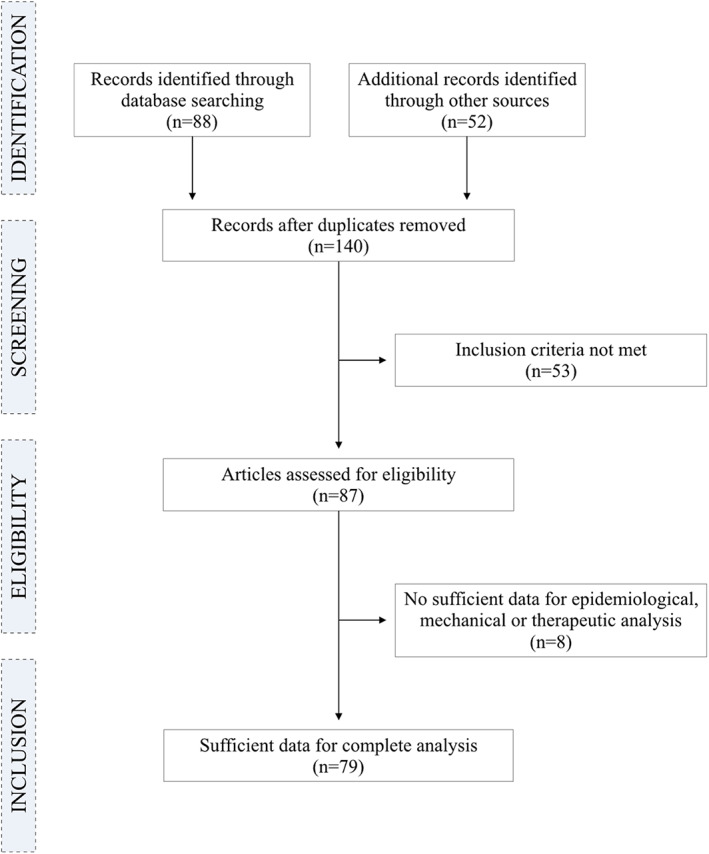
Flowchart of the systematic review.

A flowchart showing study selection is given in *Figure* 1. Data extraction led to the evaluation of 140 publications, of which 87 met the inclusion criteria, and 79 were included in the final review.^9^, ^10^, ^11^, ^12^, ^13^, ^14^, ^15^, ^16^, ^17^, ^18^, ^19^, ^20^, ^21^, ^22^, ^23^, ^24^, ^25^, ^26^, ^27^, ^28^, ^29^, ^30^, ^31^, ^32^, ^33^, ^34^, ^35^, ^36^, ^37^, ^38^, ^39^, ^40^, ^41^, ^42^, ^43^, ^44^, ^45^, ^46^, ^47^, ^48^, ^49^, ^50^, ^51^, ^52^, ^53^, ^54^, ^55^, ^56^, ^57^, ^58^, ^59^, ^60^, ^61^, ^62^, ^63^, ^64^, ^65^, ^66^, ^67^, ^68^, ^69^, ^70^, ^71^, ^72^, ^73^, ^74^, ^75^, ^76^, ^77^, ^78^, ^79^, ^80^, ^81^, ^82^, ^83^, ^84^, ^85^, ^86^, ^87^ Of these, 18 gave sufficient data for epidemiological analysis and diagnostic criteria,^9^, ^10^, ^11^, ^12^, ^13^, ^14^, ^15^, ^16^, ^17^, ^18^, ^19^, ^20^, ^21^, ^22^, ^23^, ^24^, ^25^, ^26^ 33 gave sufficient data for mechanistical analysis,^27^, ^28^, ^29^, ^30^, ^31^, ^32^, ^33^, ^34^, ^35^, ^36^, ^37^, ^38^, ^39^, ^40^, ^41^, ^42^, ^43^, ^44^, ^45^, ^46^, ^47^, ^48^, ^49^, ^50^, ^51^, ^52^, ^53^, ^54^, ^55^, ^56^, ^57^, ^58^, ^59^ and 28 gave sufficient data for therapeutic approaches.^60^, ^61^, ^62^, ^63^, ^64^, ^65^, ^66^, ^67^, ^68^, ^69^, ^70^, ^71^, ^72^, ^73^, ^74^, ^75^, ^76^, ^77^, ^78^, ^79^, ^80^, ^81^, ^82^, ^83^, ^84^, ^85^, ^86^, ^87^

### 1/Epidemiological data: Is sarcopenia a rare condition in patients with PAD?

Epidemiological analysis and diagnostic criteria were based on 17 studies following 2362 PAD patients; 31.39% (SD 7.61) were women. Mean age of patients studied was 72.42 (SD 2.84). Sarcopenia was present in 34.63% (SD 12.86) of the patients.

Sarcopenia is also described in conditions associated with PAD, such as metabolic syndrome. The metabolic syndrome—clustering of multiple metabolic abnormalities (i.e. diabetes mellitus, dyslipidemia, hypertension, or obesity)—is associated with an increased risk of cardiovascular morbidity and mortality and is highly prevalent in PAD patients.^9^

Interestingly, in a rat model of PAD, the presence of diabetes mellitus worsened the ischemia–reperfusion‐induced skeletal muscle injury, as shown by a more severe decline in mitochondrial respiration and by enhanced levels of oxidative stress and apoptosis effectors in skeletal muscles.^10^

In humans, patients living with both PAD and diabetes mellitus displayed musculoskeletal and biomechanical changes in the lower limbs. Indeed, Bartolo *et al*. recently showed a significant increase in the electromyogram muscle amplitude of PAD and diabetic patients, characteristic of less efficient lower muscle contractions and probable early fatigue.^11^

Thus, skeletal muscle wasting is exacerbated in PAD patients presenting with a metabolic syndrome. Such patients represent a particularly fragile population that requires specific attention because of prominent atherosclerotic risk factors and poor outcomes. Moreover, the accumulation of skeletal muscle alterations might enhance the prevalence of sarcopenia—particularly hidden sarcopenia—in this fragile population. Indeed, it is important to note that a large proportion of these patients might be obese and thus, might be at risk of developing sarcopenic visceral and/or subcutaneous obesity. Such potential hidden condition is resulting in a higher risk of adverse outcomes.^12^

### 2/Diagnostic criteria: How to diagnose sarcopenia and is sarcopenia a factor of poor prognosis for patients with PAD?

#### A/What is sarcopenia? Definition and diagnostic criteria

Sarcopenia is characterized by a decline in muscle mass and function with age or disease. It represents a significant burden for the patients, as it is associated with physical disability, increased hospitalization rates and mortality.^13^ It is therefore essential for healthcare professionals to better recognize this condition. In this light and during the last decade, collaborative efforts were made all over the world, for example, Europe, America, and Asia in order to reach a consensus definition and diagnosis criteria for sarcopenia. To date, it appears accepted by the different working groups on sarcopenia that sarcopenia should be defined through three main criteria: (i) low muscle strength, (ii) low muscle quantity or quality, and (iii) low physical performance. Alone, Criterion 1 is considered the most reliable, and it should guide towards the diagnosis of sarcopenia. When combined, Criteria 1 and 2 account for the certainty of the diagnosis. Last, if all three criteria are met, sarcopenia is considered severe based on the link between low physical performance and poor prognosis.

##### Muscle strength assessment

According to the European Working Group on Sarcopenia in Older People (EWGSOP), low muscle strength can be assessed with measures of grip strength, with cut‐off points under 27 kg for men and 16 kg for women. The chair stand test can be used to measure impaired lower body strength (sarcopenia is suspected if the time required to complete five consecutive rises exceeds 15 s). Finally, isometric torque methods can be used to measure muscle extension/flexion power.

##### Muscle mass/quality assessment

Low muscle quantity can be assessed by measurements of appendicular skeletal muscle mass (ASM) or skeletal muscle mass index (ASM/height^2^) using bioelectrical impedance analysis (BIA) or dual X‐ray absorptiometry (DXA). The reference values for low ASM are under 20 kg for men and 15 kg for women and under 7.0 kg/m^2^ for men and 6.0 kg/m^2^ for women for low skeletal muscle mass index. Magnetic resonance imaging (MRI) or computed tomography (CT) can also be used to appreciate muscle quantity; however, appropriate cut‐offs values are not well defined for these measurements.

##### Physical performance assessment

Low physical performance can be predicted by low gait speed (≤0.8 m/s) or low score on the short physical performance battery test (≤8 point).^14^

The data published show an important heterogeneity in the application of sarcopenic diagnostic criteria. Indeed, based on the articles in *Tables* 1 and 2, 12 articles are using a single‐measurement approach (measure of muscle mass alone in six articles, muscle strength alone in five articles and physical performance alone in one article); nine articles are using two points of measure (commonly muscle strength and physical performance), and no articles are using all three criteria (muscle strength, muscle mass, and muscle function). Where muscle mass is used, four articles focused on lumbar muscles, one on leg muscles, and three on individual psoas muscle; and values are either unindexed in five articles, indexed to tibial length square in one article, height square in one article, or even to the adjacent vertebral body in one article. These disparities make it difficult to aggregate and compare the resulting data. Further, it is unlikely that a single muscle might be used as a sentinel for sarcopenia diagnostic. These data support the need of further studies to reach a consensus allowing clear and valid diagnostic criteria for sarcopenia in PAD patients.^15^

**TABLE 1:** Association between sarcopenia and poor outcomes in PAD patients

Reference	Patients population	Number of patients	Assessment method			Outcomes measured	Main results
			Muscle strength	Muscle mass/quality	Physical performance		
Shimazoe *et al.,* 2019, Ann Vasc Surg ^26^	CLI	110	‐	Skeletal muscle areas at the L3 level (CT)	Measures of basic aspects of activities related to self‐care and mobility	3‐year overall survival; amputation‐free survival	Low activity of daily living was significantly associated with worse 3‐year overall survival and amputation‐free survival in patients with CLI and low muscle mass (defined as skeletal muscle area <114.0 cm^2^ for men and <89.8 cm^2^ for women).
Taniguchi *et al.,* 2019, Ann Vasc Dis ^25^	CLI	75	‐	Cross‐sectional area of the psoas major muscles (CT)	‐	Limb salvage and overall survival	Low muscle mass (21.4 ± 3.8 kg/m^2^ in the sarcopenic group vs. 23.5 ± 3.1 kg/m^2^ in the non‐sarcopenic group) was associated with significantly lower limb salvage rates (73% vs. 100% at 2 years, *P* < 0.05) and overall survival rates (60% vs. 87% at 3 years, *P* < 0.05)
Morisaki *et al.,* 2019, Vascular ^24^	CLI	127	‐	Low skeletal muscle mass index (CT)	Non‐ambulatory status	Overall survival	Low muscle mass (defined as skeletal muscle area <114.0 cm^2^ for men and <89.8 cm^2^ for women) was associated with significantly lower overall survival (89.7% in the CLI Frailty group vs. 60.5% in the CLI Non‐frailty group at 2 years after revascularization, *P* < 0.01)
Reeve *et al.,* 2018, J Vasc Surg ^19^	Vascular disease (AAA, carotid stenosis, PAD)	311	Dominant hand grip strength	‐	‐ N‐	Comorbidity, cardiac risk	Low muscle strength (19.7 ± 6.5 kg in the frail vs. 36.8 ± 10.3 kg in the non‐frail patients) was associated with comorbidity (based on Charlson comorbidity index with 6.4 ± 2.2 points vs. 5.2 ± 2.2 points, *P* < 0.0001) and cardiac risk (based on revised cardiac risk index with 1.8 ± 0.8 vs. 1.5 ± 0.7, *P* < 0.018)
Sugai *et al.,* 2018, Circ J ^18^	PAD	327	‐	Psoas muscle value (CT)	‐	Major adverse cardiovascular and limb events	Patients with major adverse cardiovascular and limb events had significantly lower mean psoas muscle value (41.0 ± 7.4 vs. 46.7 ± 5.7 Hounsfield unit, *P* < 0.001) than those without
Matsubara *et al.,* 2017, J Vasc Surg ^23^	CLI	114	‐	Vertebral body at the L3 level (CT)	‐	Cardiovascular event‐free survival	Low muscle mass (defined as skeletal muscle area <114.0 cm^2^ for men and <89.8 cm^2^ for women) was associated with lower cardiovascular event‐free survival rates (43.1% for patients with sarcopenia vs. 91.2% without sarcopenia at 3 years, *P* < 0.01)
Nyers *et al.,* 2017, J Vasc Surg ^21^	PAD	188	‐	Psoas‐L4 verterbal index (Cross‐sectional area of the bilateral psoas muscles and vertebral body at the L4 level) (CT)	‐	Amputation‐free survival	Muscle mass did not predict amputation‐free survival (with a psoas‐L4 vertebral index at 1.79 ± 0.55 for patients with 3 years amputation‐free survival vs. 1.78 ± 0.57 for patients without 3 years amputation‐free survival)
Matsubara *et al.,* 2015, J Vasc Surg ^22^	CLI	64	‐	Vertebral body at the L3 level (CT)	‐	Overall survival	Low muscle mass (defined as skeletal muscle area <114.0 cm^2^ for men and <89.8 cm^2^ for women) was associated with lower survival rates (23.5% for patients with sarcopenia vs. 77.5% without sarcopenia at 5 years, *P* < 0.001)
McDermott *et al.,* 2012, J Am Coll Cardiol ^17^	PAD	434	Knee extension/Isometric knee extension/Plantar flexion powerHand grip strength	Calf muscle density (CT)	‐	Comorbidities and mortality	Lower calf muscle density was associated with higher cardiovascular disease mortality. Low plantar flexion strength, low baseline leg power and poor handgrip were associated with higher all‐cause mortality (using proportional hazards analyses)
Singh *et al.,* 2010, J Vasc Surg ^20^	PAD	410	Knee extension/Isometric knee extension/Hip extension/Hip flexion power	‐	‐	Mortality	Low baseline strength for knee flexion/extension and hip extension were associated with higher all‐cause mortality in men. Poorer strength for knee flexion and hip extension were associated with higher cardiovascular mortality in men (using proportional hazards analyses)

AAA, abdominal aortic aneurysm, CLI, critical limb ischemia; CT, computed tomography; F, female; M, male; PAD, peripheral artery disease.

**TABLE 2:** Association between impaired muscle strength/function and PAD

Reference	Patients population	Number of patients/controls	Assessment method			Main results
			Muscle strength	Muscle mass/quality	Physical performance	
Kakihana *et al.,* 2017, J Vasc Surg ^35^	PAD	16/10	‐	‐	7‐m walkway embedded with a force plate test	PAD was associated with slower walk at self‐selected walking speed (88.32 ± 15.15 cm/s for PAD patients vs. 126.04 ± 16.31 cm/s for controls, *P* < 0.001) and at fast walking speed (119.90 ± 21.07 cm/s vs. 162.01 ± 21.47 cm/s for controls, *P* < 0.001); lower cadence at self‐selected walking speed (109.92 ± 12.17 step/min vs. 118.38 ± 7.28 steps/min, *P* < 0.001) and at fast walking speed (121.29 ± 11.39 steps/min vs. 135.11 ± 9.47 step/min, *P* < 0.001); and reduced peak hip flexor generation power at self‐selected walking speed (0.50 ± 0.18 W/kg vs. 1.00 ± 0.22 W/kg, *P* < 0.001) and at fast walking speed (0.78 ± 0.27 W/kg vs. 1.40 ± 0.39 W/kg, *P* < 0.001)
Schieber *et al.,* 2017, J Vasc Surg ^29^	PAD	94/16	Maximal isometric plantar flexion contractions of 10 s	‐	‐	PAD patients exhibited strength deficits, with impaired peak torque values (69.1 ± 28.7 N.m for claudicating patients vs. 98.2 ± 27.6 N.m for controls, *P* < 0.01)
Dziubek *et al.,* 2015, Maturitas ^33^	CLI	85/50	Force‐velocity parameters (peak torque, total work, average power) of the lower limb	‐	6‐min walk test	PAD was associated with lower 6‐min walk distance (349.77 ± 65.08 m for PAD patients vs. 515.86 ± 96.39 for controls, *P* < 0.0001), lower mean walk speed (3.49 ± 0.65 km/h vs. 5.15 ± 0.96 km/h for controls, *P* < 0.01), and significantly lower values of force‐velocity parameters (including peak torque, total work and average power of the knee joint) compared with the control group (*P* < 0.005)
Parmenter *et al.,* 2013, J Vasc Surg ^34^	PAD	22/−	Maximum strength/endurance testing (hip extensors, hip abductors, quadriceps, hamstrings, plantar flexors, pectoral, upper back muscles)	‐	6‐min walk test	Greater severity of PAD was associated with reduced bilateral hip extensor strength (*r* = 0.54, *P* = 0.007), whole body strength (*r* = 0.32, *P* = 0.05), shorter distance to first stop during the 6‐min walk test (*r* = 0.38, *P* = 0.05) and poorer single leg balance (*r* = 0.44, *P* = 0.03) (using univariate and stepwise multiple regression models)
Câmara *et al.,* 2012, Ann Vasc Surg ^30^	PAD	20/9	Plantar flexion/dorsiflexion movements, knee extension/flexion movements	‐	Plantar flexion/dorsiflexion movements, knee extension/flexion movements	PAD patients presented lower muscle strength in dorsiflexion (0.20 ± 0.10 N/m/kg for PAD patients vs. 0.29 ± 0.10 N/m/kg for controls, *P* < 0.01), plantar flexion (0.36 ± 0.20 N/m/kg vs. 0.53 ± 0.20 N/m/kg, *P* < 0.01) and knee flexion movements (0.50 ± 0.30 N/m/kg vs. 0.62 ± 0.10, *P* = 0.04). Also, PAD was associated with lower muscle endurance in dorsiflexion (8.0 ± 3.5 N/m/kg vs. 9.9 ± 6.6 N/m/kg, *P* = 0.01) and plantar flexion movements (20.0 ± 9.0 N/m/kg vs. 25.7 ± 10.7 N/m/kg, *P* = 0.02)
Wurdeman *et al.,* 2012, Gait Posture ^31^	PAD	30/32	Joint moments and powers at early, mid and late stance (hip and knee and ankle joints)	‐	‐	PAD was associated with reduced peak hip power absorption in midstance (−0.788 ± 0.25 W/kg for PAD patients vs.−0.950 ± 0.27 W/kg for controls, *P* = 0.017), reduced peak knee power absorption in late stance (−0.729 ± 0.21 W/kg vs.−0.899 ± 0.33 W/kg for controls, *P* = 0.02), and reduced peak ankle power generation in late stance (2.677 ± 0.45 W/kg vs. 2.998 ± 0.60 W/kg, *P* = 0.021)
Koutakis *et al.,* 2010, J Vasc Surg ^32^	PAD	20/16	Joint torques and powers at early, mid and late stance (hip, knee and ankle joints)	‐	Ambulation on a walkway	PAD patients presented significantly reduced hip power generation in late stance (0.569 ± 0.18 W/kg for claudicating patients vs. 0.706 ± 0.24 W/kg for controls, *P* = 0.03), knee power absorption in late stance (−0.580 ± 0.25 W/kg vs. −0.882 ± 0.32 W/kg, *P* = 0.0015), and ankle power generation in late stance (2.178 ± 0.51 W/kg vs. 2.957 ± 0.69 W/kg, *P* = 0.0001) Also, PAD was associated with reduced gait velocity (1.09 ± 0.13 m/s for claudicating patients vs. 1.28 ± 0.13 m/s for controls, *P* = 0.0007) and stride length (1.27 ± 0.11 m vs. 1.47 ± 0.11 m for controls, *P* < 0.001)
Koutakis *et al.,* 2010, J Vasc Surg ^36^	PAD	20/10	Joint torques and powers at early, mid and late stance (hip, knee and ankle joints)	‐	‐	PAD was associated with reduced knee power generation in early stance (0.26 ± 0.31 W/kg for claudicating patients vs. 0.62 ± 0.25 W/kg for controls, *P* < 0.05) and ankle power generation in late stance (2.05 ± 0.59 W/kg vs. 4.00 ± 0.88 W/kg for controls, *P* < 0.05)
Herman *et al.,* 2009, J Am Geriatr Soc ^37^	PAD	374/−	Hip extension/flexion, knee extension/flexion strength Walking over a force platform	‐	7‐m walking speed test 6‐min walk test Short physical performance battery	In women with PAD, weaker baseline hip and knee flexion strength were associated with faster average annual decline in usual‐paced 4‐m walking velocity (*P* trend < 0.001 and *P* trend = 0.02 respectively) and in short physical performance battery test (*P* trend = 0.019 and *P* trend = 0.01, respectively)
McDermott *et al.,* 2008, J Am Geriatr Soc ^28^	PAD	424/271	Isometric knee extension/plantar flexion strength Handgrip strength Knee extension power	‐	6‐min walk test 4‐m walking velocity test	Lower arterial brachial index values were associated with lower plantar flexion strength (*P* trend = 0.04) and lower knee extension power (*P* trend < 0.001)
Kuo *et al.,* 2008, J Gerontol A Biol Sci Med Sci ^38^	PAD	206/1592	Isokinetic dynamometer	‐	20‐ft timed walk test	PAD associated with weak leg force, low gait speed and functional dependence (based on multiple logistic regression analyses)

#### B/Sarcopenia in PAD: a factor of poor prognosis

Sarcopenia has been associated with multiple adverse events such as physical limitation, poor quality of life, and mortality and was shown to predict patients' prognosis and outcome following vascular procedures.^16^ Similarly, recent data have revealed a link between sarcopenia, PAD and high comorbidity. In a 48‐month longitudinal study, a reduction in calf muscle density, lower limb extension/flexion power, and hand grip strength was shown to be associated with an increase in mortality in 434 PAD patients.^17^ Interestingly, this conclusion was also reached when only one parameter of sarcopenia was measured (muscle quantity^18^ or muscle strength^19^). Indeed, in a retrospective study of 327 patients with PAD followed for up to 30 months, Sugai *et al*. found an independent link between low psoas muscle area and major adverse cardiovascular and limb events. Likewise, after a 9‐month follow‐up, Reeve *et al*. highlighted an association between lower grip strength and elevated comorbidity and cardiac risk in patients with vascular diseases, including PAD. Noteworthy, in a study realized on 410 patients with PAD followed for 60 months, poor leg extension/flexion power was reported to predict mortality in men but not in women.^20^ This result might be explained by the larger proportion of men studied. Finally, a recent study following patients with PAD for 72 months showed no difference on overall survival between patients with high or low muscle mass, assessed through comparison of psoas‐L4 vertebral index.^21^ Together, these results indicate that muscle strength rather than muscle mass is a poor prognosis factor in PAD. This might be attributable to the fact that muscle mass measurements are extremely susceptible to bias, supporting the need for homogenized technic used, muscle analysed, and cut‐off values of low muscle mass.

Sarcopenia was also linked with CLI and high mortality rates. Indeed, in 2015, and subsequently in 2017, Matsubara *et al*. reported that sarcopenia was associated with higher cardiovascular events and lower survival, in 64 and 114 patients suffering from CLI, respectively.^22^, ^23^ Recent papers confirmed this association in retrospective studies including patients with CLI, where low skeletal muscle mass was predictive of a worse overall survival^24^, ^25^, ^26^ (*Table* 1).

### 3/Mechanistical analysis: how does sarcopenia affect skeletal muscle?

Mechanistical analysis was based on five animal studies [10; 43–44; 48; 58] and 29 clinical reports. This allowed the analysis of the following:

- muscle strength and function,^27^, ^28^, ^29^, ^30^, ^31^, ^32^, ^33^, ^34^, ^35^, ^36^, ^37^, ^38^ based on 1352 PAD patients [26.49% (SD 17.32) women], aged 67.67 (SD 5.14) years;
- muscle histology,^39^, ^40^, ^41^, ^42^ based on 192 PAD patients [9.2% (SD 11.22) women], aged 64.3 (SD 0.99) years;
- oxidative stress,^43^, ^44^, ^45^, ^46^, ^47^ based on 69 PAD patients [16.96% (SD 8.10) women], aged 63.17 (SD 1.43) years;
- mitochondriopathy [43; 47–53], based on 153 PAD patients [29.39% (SD 28.27) women], aged 63.50 (SD 1.83) years;
- inflammation,^54^, ^55^, ^56^ based on 900 PAD patients [40.77% (SD 3.71) women], aged 74.88 (SD 2.76) years;
- signalling pathways [10; 57–59], based on 51 PAD patients [34.45% (SD 32.23) women], aged 72.25 (SD 5.25) years.

### Muscle dysfunction in PAD: the missing link to better understand the common mechanisms of sarcopenia and PAD pathophysiology

**FIGURE 2:**
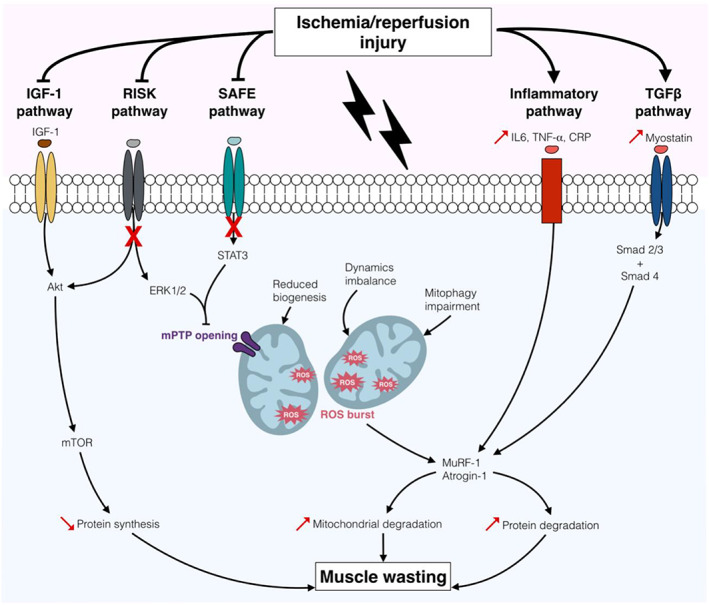
Major signalling pathways associated with sarcopenia and peripheral arterial disease (PAD). In the context of PAD, ischemia/reperfusion (I/R) injury induces a decrease in mitochondrial biogenesis, dynamics, and mitophagic activities, resulting in reactive oxygen species (ROS) burst and consecutive oxidative stress. Additionally, elevated levels of IL6, TNF‐α, and CRP are responsible for the activation of the inflammatory pathway. Ultimately, I/R‐induced oxidative stress and inflammation enhance the activity of the atrophy‐related ubiquitin ligases MuRF‐1 and atrogin‐1 and the degradation of mitochondria and proteins. I/R is also associated with defective stimulation of the muscle synthesis PI3K/Akt/mTOR pathway, notably via lower activity of the IGF‐1 and RISK pathways. Moreover, alteration of the protective pathways RISK and SAFE lead to persistent mitochondrial permeability transition pore (mPTP) opening, reduction in mitochondrial calcium retention capacity, and aggravation of mitochondrial dysfunction. Further, during I/R, myostatin overexpression results in enhanced activity of the muscle degradation pathway TGFβ.

Patients with lower extremity PAD present various skeletal muscle defects, such as weak baseline strength, functional decline, and abnormal muscle histology. Although sarcopenia is currently defined by its clinical manifestations, all etiological factors identified so far—including excessive oxidative stress production, skeletal muscle mitochondrial impairments, high inflammation, and altered muscle kinetic process—are present in PAD and emphasizing skeletal muscle injuries (*Figure* 2).

#### A/Impaired muscle strength and function

Peripheral arterial disease pathophysiology is characterized by metabolic and structural myopathic changes in skeletal muscles, responsible for the decline in strength and function.^27^ Lower extremity ischemia was shown to specifically impair proximal and distal leg muscles. Indeed, in a study of 424 patients with PAD, plantar flexion and knee extension strength was significantly lower when compared with age‐matched controls.^28^ Similarly, analysis of strength and endurance of hip, knee, ankle, and plantar muscles of patients with and without PAD revealed an association between PAD and weaker leg muscles.^29^, ^30^, ^31^, ^32^ PAD physiopathology is thus characterized by a failure of specific muscles that are necessary for normal walking.

Accordingly, the loss of muscle strength appears to play a central role in the functional defects observed in patients. In the chronic stage of PAD, values of force‐velocity parameters of the lower limbs and 6‐min walk capacity were significantly lower compared with the control group.^33^ Interestingly, the impairment in force and mobility was also seen in earlier stages of PAD, as shown by three independent studies. In the first study, 22 patients with PAD underwent 6‐min walking speed testing, maximum strength/endurance testing of lower limb muscles, as well as performance‐based testing of muscle function. Patients with PAD presented overall body disability, muscle weakness, and reduced physical performance.^34^ The other two studies highlighted a gait impairment in patients with PAD, using a walkway embedded with a force plate.^35^, ^36^ Moreover, in a study of 374 patients with PAD, the correlation between poor strength/mobility and lower limb ischemia was found in women, but not men, probably because of their higher baseline strength.^37^

It is important to note that in a total of 11 studies associating low muscle strength and/or function and/or quality and PAD, none used the term sarcopenia. Yet a sarcopenic diagnosis represents a turning point for patients, families, and clinicians. Indeed, beyond aggravating patients' prognosis, sarcopenia constitutes a great burden, as it participates to the isolation and dependence of patients with PAD^38^ (*Table* 2).

#### B/Impaired muscle histology

The morphological and physiological consequences of the denervation–reinnervation process that occur in PAD were assessed in 26 patients with PAD based on *gastrocnemius* cross‐sectional area values and peak treadmill walking time. Overall, PAD was characterized by a general decline in type II muscle fibres number and size, responsible for the decay in general muscle strength.^39^ Subsequent studies focusing on myofibre morphometrics of PAD *gastrocnemius* revealed significant changes in muscle quality, including lower diameter and density, rounder myofibres, and a fast‐to‐slow switch resulting in a predominance of type I fibres, as demonstrated by cross‐sectional area analysis.^40^, ^41^, ^42^

Thus, the genesis of sarcopenia in PAD patients might be explained by the progressive loss of motoneurons included in type II motor units, which remains uncompensated despite reinnervation of muscle fibres by adaptive sprouting.

#### C/Oxidative stress

An imbalance between pro‐oxidant and antioxidant activities (also called oxidative stress) is responsible for reactive oxygen species (ROS) accumulation, mitochondrial respiratory chain dysfunctions, and oxidative damage of DNA. In PAD, ischemic lesions generate extensive oxidative stress. Accordingly, murine models of PAD displayed reduced antioxidant enzymes mRNA levels, increased ROS production,^43^ increased oxidative stress, and impaired mitochondrial respiration, notably with reduced electron transport chain complexes I, III, and IV activities in ischemic muscles.^44^

Similarly, human studies reported altered antioxidant enzyme activities, reduced electron transport chain complexes I, III, and IV activities and impaired mitochondrial respiration in patients with PAD.^45^ PAD was also associated with significant oxidative stress and ROS production.^46^ Interestingly, the extend of oxidative damage in PAD *gastrocnemius* was shown to be associated with advanced disease stage and lower myofibre cross‐sectional area.^47^

Taken together, these data suggest the pathological implication of excessive oxidative stress in myofibres damage and PAD.

#### D/Mitochondriopathy

With disease and/or advancing age, mitochondrial dysfunctions accumulate, thus disrupting vital mitochondrial‐dependent activities.^47^ Accordingly, studies in murine models of CLI revealed mitochondrial DNA damages in ischemic aged muscles, lower mitochondrial respiration, declined mitochondrial biogenesis, impaired calcium retention capacity and muscle atrophy, and muscle contractile deficits.^43^, ^48^

Human investigations regarding mitochondrial dysfunction in PAD showed an increase in skeletal muscle mitochondrial DNA injuries^49^ and abnormal mitochondrial respiratory activity of ischemic muscles^50^ compared with controls. Interestingly, Koutakis *et al*. investigated the potential role of the muscle specific intermediate filament desmin in PAD pathophysiology and linked abnormal desmin accumulation with low mitochondrial respiration.^51^ Moreover, immunohistochemical analysis of muscle biopsies revealed accumulation of microtubule‐associated protein light chain 3 (LC3)—an autophagic marker—in the area depleted of mitochondria in PAD myofibres, thus suggesting an association between PAD and aberrant mitophagy process.^52^ Last, a recent work has stressed a unique and severe mitochondriopathy touching patients with CLI. Indeed, besides the reduced mitochondrial oxidative capacity and respiratory activity also observed in patients with PAD, CLI was shown to be characterized by deficits in permeabilized myofibre mitochondrial function and decreased abundance of mitochondria‐associated mRNAs and proteins.^53^

Overall, mitochondriopathy is thought to be a major contributor to sarcopenia and PAD, notably with significant disruption of mitochondrial biogenesis, dynamics, and mitophagy.

#### E/Inflammation

Systemic inflammation is considered an important pathophysiological mechanism in PAD and likely contributes to skeletal muscle wasting. Accordingly, several vascular inflammatory markers such as IL6, IL1 receptor antagonist, fibrinogen, and CRP were found elevated in PAD patients compared with controls subjects.^54^ Further studies revealed an association between higher levels of these markers of inflammation and poorer 6‐min walk distance and overall performance (combining walking speed, balance, and chair rises exercises),^55^ lower calf strength, and more adverse calf muscle characteristics.^56^

It is therefore possible that sarcopenia in PAD patients finds its origin in altered inflammatory process, likely mediated by the dysregulation of multiple cytokine factors.

#### F/Impaired signalling pathways

##### IGF‐1 synthesis pathway

The IGF‐1/PI3K/Akt/mTOR signalling pathway is a key player in muscle growth, stimulating protein synthesis, and satellite cell proliferation in muscle, all together while simultaneously suppressing pathways responsible for protein degradation. Regarding the exact role played by this synthesis pathway in PAD, data are scarce. In 2004, Tuomisto *et al*. reported an up‐regulation of the anabolic factors IGF‐1 and IGF‐2 in atrophic and regenerating ischemic myocytes of patients with CLI.^57^ The IGF system could promote skeletal muscle survival, regeneration and angiogenesis under chronic ischemia, notably via the VEGF pathway.

##### RISK and SAFE protective pathways

The RISK and SAFE pathways play essential roles in the reduction of ischemia/reperfusion (I/R) injuries, as they participate to muscle regeneration and mitochondrial integrity. Though this phenomenon is well documented in the field of cardiology, very little is known during PAD. In our rat model of PAD exposed to 3 h of ischemia followed by 2 h of reperfusion, the RISK and SAFE pathways were inefficiently activated, leading to mitochondrial dysfunctions, increased oxidative stress and apoptosis.^10^

##### Impaired muscle degradation pathways

Several members of the TGFβ superfamily play a key role in protein degradation, among them, myostatin is well known for the extreme hyper muscularity of myostatin knock‐out mice and conversely, for the muscle atrophy of mice overexpressing myostatin. In mice models of PAD, silencing of myostatin led to *gastrocnemius* hypertrophy and improved running performance.^58^

In humans, very little is known about the role of the TGFβ superfamily in PAD pathology. A recent research conducted by Ha *et al*. showed that TGFβ1 expression increased with advancing PAD severity.^59^ Overall, myostatin is thought to be an important factor in the pathophysiology of sarcopenia and PAD.

Overall, evidence seem to indicate an imbalance between protein synthesis and degradation leading to reduced or impaired anabolic pathway and, in a broader sense, impaired muscle function and strength in PAD.

#### 4/Therapeutic approaches: can we reverse the sarcopenic condition in patients with PAD?

Therapeutic analysis was based on seven animal studies [61–63; 83–86] and 21 clinical reports, Among the clinical reports, 19 were observational trial studies [64–68; 70–82; 87], and two were prospective studies [60; 69]. In total, 884 patients followed an exercise therapy, and 18 received an angiogenesis treatment; 30.84% (SD 17.74) were women. Mean age of patients studied was 66.85 (SD 3.96).

### Treating sarcopenia in PAD

Lower limb revascularization surgery is the treatment of choice for patients suffering from CLI, enabling blood flow restoration and limb salvage, while reversing some sarcopenic features. Indeed, in a prospective study following 18 patients with CLI, surgical revascularization improved muscle strength, 6‐min walk distance, bodily pain and overall quality of life.^60^ However, these muscular parameters are not currently tested in routine clinical practice, and therefore, we do not know whether ischemia‐related muscle impairment are normalized or whether sequela remain. Nevertheless, additional therapeutic approaches like exercise training or angiogenesis therapies seems useful to further sustain functional muscular improvement and to preserve patients' quality of life.

#### A/Exercise training

##### Molecular and cellular effects

In a mouse model of chronic CLI, moderate exercise consisting in a 3‐week treadmill training up to five times per week enhanced mitochondrial biogenesis and antioxidant enzymes mRNA levels, restored mitochondrial respiration and calcium retention capacity and reduced tissue damages, while generating low amount of oxidative stress.^61^ On the other hand, Hain *et al*. showed that repeated cycles of electrical stimulation (mimicking exercise) resulted in increased NF‐κB activity and muscle fibre atrophy.^62^ Further, 2‐week treadmill exercise was shown to have consequences on skeletal muscle mRNA expression in PAD models, notably by down‐regulating skeletal muscle regeneration markers.^63^ These results might indicate that exercise intensity could be associated with adverse effects on skeletal muscle function and might also underscore the beneficial systemic effects of exercise.

In humans, studies show a significant increase of the inflammatory markers ICAM‐1, VCAM‐1, TNF‐α, and IL6 directly following one treadmill exercise.^64^, ^65^ However, a reduction in the inflammatory process was shown in PAD patients following either a 8‐week supervised training program^66^ or a 12‐week homebased exercise training.^67^ Last, 6 to 12 months of progressive resistance training was shown to increase type I and type II skeletal muscle fibre areas and capillary density.^68^

##### Functional and vascular effects

In humans, the impact of exercise on PAD was assessed in prospective studies following training sessions for 4 weeks up to 12 months.

##### 4‐week program

After a 4‐week rehabilitation program consisting in walking exercises, selective muscle strengthening, and sports, patients suffering from PAD showed significant improvement in walking distance.^69^

##### 8‐week program

Patients following 8 weeks of maximal strength training alone,^70^ or combined with plantar flexion endurance training,^71^ presented increased leg strength, force development, and walking performance.

##### 12‐week program

A 12‐week homebased exercise training was shown to positively influence vascular function (microcirculation of the lower extremities measured by calf muscle haemoglobin oxygen saturation) and endurance (6‐min walk distance, peak walking time, and daily ambulatory activity) in PAD patients.^67^

The effects of a 12‐week treadmill‐walking program on PAD were analysed in several independent studies. Notably Wang *et al*. demonstrated the importance of this training program in improving endurance (walking capacity) and strength (peak force, peak torques in plantar flexion) in patients with PAD.^72^ The improvements in endurance (pain‐free walking distance, 6‐min walking distance, and peak exercise performance) were accompanied by a large decline in bilateral thigh lean mass^73^ or by a significant increase in the number of denervated calf muscle fibres.^74^ Here, regions remote to the ischemic lesion are mostly affected by a decline in muscle mass and muscle quality, possibly caused by sensory and motor nerve dysfunction during exercise. Further, this training program was shown to improve both muscular function (i.e. increased pain‐free walking distance) and endothelial function (i.e. increased flow‐mediated dilatation) in PAD patients.^75^ Interestingly, a 12‐week treadmill walking exercise was found more effective than a 12‐week strength training program in term of exercise performance.^76^

##### 12‐week vs. 24‐week program

A supervised treadmill walking exercise program was shown to improve exercise performance and overall functional status after 12 weeks, with continued improvement after 24 weeks.^76^, ^77^

##### 24‐week program

The effects of two training program were assessed in patients suffering from PAD. Improvements in 6‐min walk and treadmill walking performance, brachial artery flow‐mediated dilatation, and overall quality of life were observed in the group following supervised treadmill training; whereas climbing ability, treadmill performance, and quality of life were ameliorated in the group following resistance training, when compared with controls.^78^

##### 6‐month to 12‐month program

Two studies demonstrated the functional and vascular benefits of a 6‐month supervised walking exercise in PAD. Indeed, Andrew *et al*. observed improvements in peripheral circulation, walking economy, and cardiopulmonary function^79^; while Schieber *et al*. observed improvements in walking distance, muscle strength, and gait biomechanics, as well as overall quality of life.^80^ Moreover, in medium to longer term (i.e. 6 to 12 months follow‐up), progressive resistance training was shown to have beneficial effects on walking ability^81^ and muscle strength.^82^

In brief, exercise induces (i) molecular adaptations, notably with modifications of the transcript levels of mitochondrial biogenesis, antioxidant, oxidative phosphorylation enzymes, and reduction of the inflammatory process; (ii) cellular adaptations with enhanced mitochondrial plasticity, muscle fibre areas, and capillarization; and (iii) functional and vascular adaptations with enhanced functional status and improved microvascular circulation of the lower extremities in PAD (*Table* 3). Regular physical activity is highly recommended to prevent or reduce the onset of adverse effects occurring with PAD.

**TABLE 3:** Effects of exercise on sarcopenia associated with PAD in experimental and clinical studies

Reference	Population	Number studied (symptomatic/controls)	Exercise therapy		Outcomes measured			Main results
			Exercise program	Duration	Muscle strength	Muscle mass/quality	Physical performance	
Nagase *et al*., 2017, PLoS One ^63^	Mice, PAD	6/4	Treadmill training	2 weeks (twice a week)	‐	Quantitative analysis of mRNA levels	‐	Treadmill training significantly reduced the mRNA expression of skeletal muscle regeneration markers (*P* < 0.05) compared with the non‐exercised PAD group
Lejay *et al.,* 2017, Front Physiol ^61^	Mice, CLI	10/10	Treadmill training	3 weeks (5 times per week)	‐	Histological analysis	Functional score	Treadmill training reduced tissue damage (with a score of 1.9 for the exercised group vs. 4.0 for the non‐exercised group at day 30, *P* < 0.01), enhanced muscle function (with a score of 1.4 for the exercised group vs. 2.8 for the non‐exercised group at day 30, *P* < 0.01), stimulated mitochondrial biogenesis and anti‐oxidant defences
Hain *et al.,* 2011, Am J Physiol Regul Integr Comp Physiol ^62^	Rats, PAD	Ns	Electrical stimulation causing repeated muscle contractions and mimicking exercise	5 days	‐	Fibre cross‐sectional area	‐	Repeated cycles of muscle contraction decreased the mean fibre cross‐sectional area by 35% (1834 ± 219.9 μm^2^ in the exercised group vs. 2834 ± 132.5 μm^2^ in the non‐exercised group, *P* < 0.05)
Schieber *et al.,* 2019, J Vasc Surg ^80^	Human, PAD	47/−	Supervised walking exercise	6 months (3 times per week)	Plantar flexor strength	‐	Walking distance, gait biomechanics	Supervised walking exercise improved muscle strength, walking distance and gait biomechanics
Vun *et al.,* 2016, J Vasc Surg ^73^	Human, PAD	36/−	Supervised treadmill exercise program	12 weeks (twice a week)	‐	Whole‐body dual‐energy X‐ray absorptiometry	Pain‐free walking distance 6‐min walking distance	Supervised treadmill exercise improved pain‐free walking distance (213 ± 93 m after 12 weeks vs. 165 ± 78 m at baseline, *P* = 0.001) and 6‐min walk distance (421 ± 68 m after 12 weeks vs. 395 ± 78 m at baseline, *P* = 0.004)
Gardner *et al.,* 2014, J Am Heart Assoc ^67^	Human, PAD	60/−	Step‐monitored home walking to mild‐to‐moderate claudication pain	12 weeks (3 times per week)	‐	‐	6‐min walking distance Walking speed	Home walking exercise improved 6‐min walk distance (372 ± 119 m after the 12‐week test vs. 328 ± 108 m at pre‐test, *P* < 0.001), peak walking time (490 ± 350 s vs. 380 ± 274 s at pre‐test, *P* < 0.001), and daily ambulatory activity notably with improvement in average cadence (11.8 ± 3.0 strides/min vs. 11.1 ± 2.7 strides/min, *P* < 0.01)
Januszek *et al*., 2014, J Cardiol ^75^	Human, PAD	67/−	Supervised treadmill training	12 weeks (3 times per week)	‐	‐	Maximal walking time	Treadmill training improved maximal walking time (+90%, *P* < 0.001) and flow‐mediated dilatation (+43%, *P* < 0.001) in PAD patients in comparison to baseline
Pilz *et al.,* 2014, Wien Klin Wochenschr ^82^	Human, PAD	42/−	Supervised exercise training on strength (couch pedal ergometer work on lower legs) and endurance (walk sessions)	6 months (twice a week)	Pushing power Pulling power Tip‐toe standing power	‐	Pain‐free walking distance Walking‐speed	Combined exercise program improved walking distance (568.9 ± 461.5 m after 6 months vs. 446.3 ± 276.6 m at baseline, *P* < 0.05), walking speed (4.39 ± 1.08 km/h vs. 4.17 ± 0.85 km/h at baseline, *P* < 0.05), pushing power (662.4 ± 530.4 J vs. 348.6 ± 270.3 J, *P* < 0.01), pulling power (96.4 ± 51.5 J vs. 58.7 ± 37.7 J, *P* < 0.0001), and tiptoe standing power (83.5 ± 48.6 repetitions vs. 49 ± 21.5 repetitions, *P* < 0.0001)
		52/−		12 months (twice a week)				Combined exercise program further improved walking distance (647.8 ± 496.3 m after 12 months vs. 500.2 ± 427.9 m at baseline, *P* < 0.001), walking speed (4.53 ± 0.80 km/h vs. 4.03 ± 0.90 km/h at baseline, *P* < 0.0001), pushing power (637.8 ± 407.1 J vs. 337.2 ± 232.9 J, *P* < 0.001), pulling power (97.5 ± 59.8 J vs. 55.6 ± 38.8 J, *P* < 0.0001), and tiptoe standing power (84.9 ± 69.3 repetitions vs. 39.8 ± 15.3 repetitions, *P* < 0.0001)
Parmenter *et al.,* 2013, J Am Geriatr Soc ^81^	Human, PAD	7/−	High‐intensity progressive resistance training (weight lifting)	6 months (3 times per week)	‐	‐	6‐min walking distance	Progressive resistance training increased 6‐min walking distance (381.8 ± 151.6 m after 24 weeks of training vs. 321.9 ± 109.1 m at baseline, *P* = 0.02)
Mosti *et al.,* 2011, Scand J Med Sci Sports ^71^	Human, PAD	10/−	Leg press maximal strength training and plantar flexion endurance training	8 weeks (3 times per week)	Leg press maximal force Rate of force development	‐	Plantar flexion endurance	Exercise training improved muscle strength, notably with increased rates of force development (3675 ± 1315 N/s post‐test vs. 1943 ± 1027 N/s pre‐test, *P* < 0.01) and leg press maximal strength (152 ± 33 kg vs. 110 ± 24 kg, *P* < 0.01); but also walking distance (1203 ± 451 m vs. 1099 ± 463 m, *P* < 0.01)
Cousin *et al.,* 2011, Ann Phys Rehabil Med ^69^	Human, PAD	31/−	Walking sessions, selective muscle strengthening, general physical exercise	4 weeks (5 days per week)	Ankle plantar and dorsal flexors strength Concentric contractions at the angular velocity of 30°/s, 120°/s and 180°/s for muscle fatigue	‐	Walking distance on a treadmill <400 m	Rehabilitation program improved walking distance (977.4 ± 854.2 m upon completing the program vs. 282.4 ± 239.8 m at baseline, *P* < 0.0001)
Saetre *et al.,* Angiology, 2011 ^66^	Human, PAD	29/−	Supervised exercise training	8 weeks (twice a week)	‐	Quantitative analysis of plasma inflammatory levels	Pain‐free walking distance, maximal walking distance	Exercise training reduced the plasma levels of E‐selectin (45.5 before training to 40.4 ng/ml after training, *P* = 0.013) and ICAM‐1 (342.0 to 298 ng/ml) in PAD patients. Both walking distance increased after exercise training (*P* < 0.01)
Wang *et al.,* 2010, Scand J Med Sci Sports ^70^	Human, PAD	10/−	Maximal strength training (dynamic leg press)	8 weeks (3 times per week)	Leg press force Rate of force development	‐	Walking economy test	Maximal strength training improved rates of force development (2901 ± 1848 N/s after the 8‐week training program vs. 1368 ± 893 N/s in the control period, *P* < 0.05), maximal strength (148 ± 33 kg vs. 114 ± 25 kg) and walking time to exhaustion (1095 ± 426 s vs. 1009 ± 448 s, *P* < 0.05)
McDermott *et al.,* 2009, JAMA ^78^	Human, PAD	156/−	Supervised treadmill walking training *vs*.resistance training	24 weeks (3 times per week)	‐	‐	6‐min walk performance, short physical performance battery, treadmill walking performance, walking impairment questionnaire, overall physical functioning score	Supervised treadmill walking training improved 6‐min walk performance (by 35.9 m, *P* < 0.001), maximal treadmill walking time (by 3.44 min, *P* < 0.001) and overall quality of life (*P* = 0.02) compared to untrained controls. Resistance training increased maximal treadmill walking time (by 1.90 min, *P* = 0.009), stair climbing (*P* = 0.03) and overall quality of life (*P* = 0.04)
Wang *et al.,* 2006, Clin J Sport Med ^72^	Human, PAD	17/−	Supervised treadmill walking training	12 weeks (3 times per week)	Calf‐muscle strength and endurance	‐	Walking capacity	Supervised treadmill‐walking program improved peak torque at 30 degrees/s (175 ± 40 N/m post‐training vs. 159 ± 32 N/m at pre‐training, *P* < 0.01), mean peak force (358 ± 87 N vs. 314 ± 68 N, *P* < 0.001), and mean power (80 ± 26 W vs. 66 ± 19 W, *P* < 0.001). This training program also increased pain‐free walking time (382 ± 261 s vs. 137 ± 70 s, *P* < 0.001) and maximal walking time (696 ± 191 s vs. 314 ± 138 s, *P* < 0.001)
Signorelli *et al.,* 2003, Vasc Med ^65^	Human, PAD	20/20	Treadmill test	1 session	‐	Quantitative analysis of plasma inflammatory levels	‐	One treadmill exercise session increased plasma levels of ICAM‐1 (317 ± 4 at rest to 421 ± 10 ng/ml after exercise), VCAM‐1 (485 ± 14 to 576 ± 16), TNF‐α (14 ± 3 to 27 ± 5) and IL6 (12 ± 1 to 16 ± 2) in PAD patients
McGuigan *et al.,* 2001, J Gerontol A Biol Sci Med Sci ^68^	Human, PAD	11/−	Progressive resistance training	6 months (3 times per week)	Leg press strength Calf press strength	Biopsies from gastrocnemius muscles	‐	Progressive resistance training improved the 10‐repetition maximum loading leg (by 155%) and calf (by 126%) press strength in the trained subjects, at 24 weeks Training also increased type I (3442 ± 981 μm^2^ after training vs. 2695 ± 867 μm^2^ at pre‐training, *P* < 0.05) and type II muscle fibre area (4273 ± 1113 μm^2^ vs. 3421 ± 1148 μm^2^, *P* < 0.05)
Brevetti *et al.,* 2001, Clin Hemorheol Microcirc ^64^	Human, PAD	21/18	Maximally tolerated treadmill exercise	1 session	‐	Quantitative analysis of plasma inflammatory levels	‐	One treadmill exercise session increased plasma levels of ICAM‐1 (285 ± 15 at rest to 317 ± 16 ng/ml after exercise, *P* < 0.01) and VCAM‐1 (671 ± 45 to 751 ± 47 ng/ml, *P* < 0.05) in PAD patients, while no modifications were observed in controls
Gardner *et al.,* 2000, J Gerontol ^79^	Human, PAD	63/−	Supervised walking exercise	6 months (3 times per week)	‐	‐	Walking economy	Exercise training improved walking economy by 10% (*P* < 0.05) compared with the untrained group
Hiatt *et al.,* 1996, J Appl Physiol ^74^	Human, PAD	26/−	Treadmill walking exercise	12 weeks (3 times per week)	‐	Biopsies from gastrocnemius muscles	Peak exercise performance	Treadmill training was associated with improved exercise performance despite increased denervated fibres (7.6 ± 5.4 before exercise to 15.6 ± 7.5% after exercise, *P* < 0.05)
Regensteiner *et al.,* 1996, J Vasc Surg ^77^	Human, PAD	29/−	Supervised treadmill walking training	12 weeks (3 h per week) or 24 weeks (3 h per week)	‐	‐	Functional status (questionnaires on walking ability, habitual physical activity level, and physical/social functioning, well‐being, overall health); monitored activity levels	Exercise training improved functional status and monitored activity level (*P* < 0.05) after 12 weeks and to a greater extend after 24 weeks
Hiatt *et al.,* 1994, Circulation ^76^	Human, PAD	29/−	Supervised treadmill walking training *vs*.strength training (resistive training of five muscle groups of each leg)	12 weeks (3 h per week) or 24 weeks (3 h per week)	‐	‐	Peak exercise performance	Patients in the 12 weeks treadmill training program had higher increase in peak walking time and higher improvement in peak oxygen consumption and onset of claudication pain compared with patients in the strength training program; with further improvements over 24 weeks of training

CLI, critical limb ischemia; Ns, not specified; PAD, peripheral artery disease.

##### Angiogenesis

PAD and CLI are characterized by vascular dysfunction, reduced microvascular flow and altered angiogenesis process. On this basis, therapeutic angiogenesis represents a promising approach in the restoration of blood flow and treatment of ischemic lesions. In mouse models of CLI, angiogenic therapy consisting in bone marrow cells injections resulted in reduced limb necrosis and muscle impairment, enhanced *gastrocnemius* and quadriceps muscle mass, and blood flow regeneration, compared with untreated ischemic animals.^83^, ^84^, ^85^ Interestingly, in a murine hindlimb ischemic model, injections of donepezil—an anti‐Alzheimer drug—upregulated angiomyogenesis factors (VEGF, HIF‐1α, and Akt) and reduced skeletal muscle atrophy.^86^

Data in humans did not confirm the potential benefice of angiogenesis therapy in PAD, notably showing similar exercise performance and survival rates.^87^

## Concluding remarks and perspectives

**FIGURE 3:**
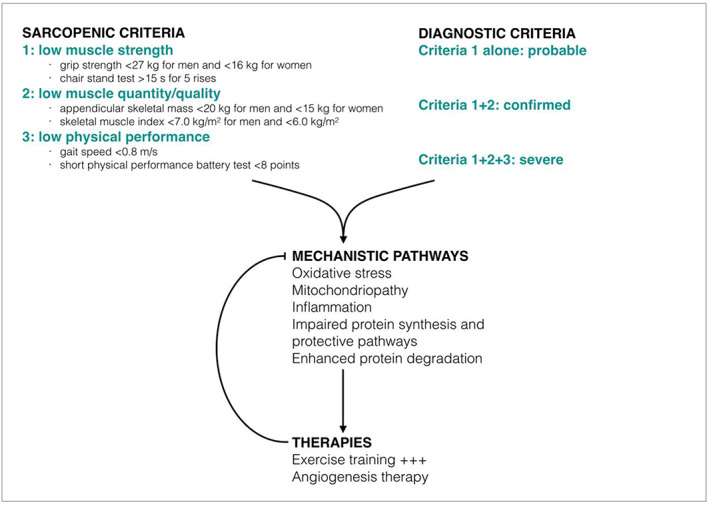
Sarcopenia and PAD: Diagnostic criteria, mechanistic pathways, and current therapies.

With more than 200 million individuals affected worldwide, lower extremity PAD is a major issue for public healthcare. The morbidity and mortality rates are alarmingly high, especially in patients also presenting with sarcopenia. The mechanistic link between sarcopenia and PAD remains to be investigated but likely involves oxidative stress, mitochondrial dysfunction, inflammation and impaired muscle synthesis, and degradation pathways. Although difficult, the diagnosis of sarcopenia is crucial for PAD patients' care, as it determines prognosis, quality of life, and possible treatments. Indeed, targeting the muscular defects through exercise training could reverse the sarcopenic features observed in patients suffering from PAD and thus, ameliorate their quality of life and overall prognosis. Further, although therapeutic data are largely contradictory, complementary pharmacologic strategies focused on muscle mitochondrial dysfunction through oxidative stress, inflammation, and/or angiogenesis modulation should be further investigated in view of their potential usefulness as new innovative therapeutic approaches against sarcopenia (*Figure* 3).

### Ethical statement

The authors certify that they comply with the ethical guidelines for authorship and publishing in the *Journal of Cachexia, Sarcopenia and Muscle*.^88^

## Conflict of interest

None declared.
